# Retracted: Immunomodulatory Effect of *Lycium barbarum* Polysaccharides against Liver Fibrosis Based on the Intelligent Medical Internet of Things

**DOI:** 10.1155/2023/9852810

**Published:** 2023-01-03

**Authors:** Journal of Healthcare Engineering

*Journal of Healthcare Engineering* has retracted the article titled “Immunomodulatory Effect of *Lycium barbarum* Polysaccharides against Liver Fibrosis Based on the Intelligent Medical Internet of Things” [1] due to concerns that the peer review process has been compromised.

Following an investigation conducted by the Hindawi Research Integrity team [2], significant concerns were identified with the peer reviewers assigned to this article; the investigation has concluded that the peer review process was compromised. We therefore can no longer trust the peer review process, and the article is being retracted with the agreement of the Chief Editor.

The authors agree to the retraction; author Wei Li could not be reached by the publisher using the e-mail address provided with the article submission.
